# The Spectrum of Clinical, Immunological, and Molecular Findings in Familial Hemophagocytic Lymphohistiocytosis: Experience From India

**DOI:** 10.3389/fimmu.2021.612583

**Published:** 2021-03-05

**Authors:** Snehal Shabrish, Madhura Kelkar, Reetika Malik Yadav, Umair Ahmed Bargir, Maya Gupta, Aparna Dalvi, Jahnavi Aluri, Manasi Kulkarni, Shweta Shinde, Sneha Sawant-Desai, Priyanka Kambli, Gouri Hule, Priyanka Setia, Neha Jodhawat, Pallavi Gaikwad, Amruta Dhawale, Nayana Nambiar, Vijaya Gowri, Ambreen Pandrowala, Prasad Taur, Revathi Raj, Ramya Uppuluri, Ratna Sharma, Pranoti Kini, Meena Sivasankaran, Deenadayalan Munirathnam, Ramprasad Vedam, Pandiarajan Vignesh, Aaqib Banday, Amit Rawat, Amita Aggarwal, Ujjal Poddar, Meenakshi Girish, Abhijit Chaudhary, Abhilasha Sampagar, Dharani Jayaraman, Narendra Chaudhary, Nitin Shah, Farah Jijina, S. Chandrakla, Swati Kanakia, Brijesh Arora, Santanu Sen, Madhukar Lokeshwar, Mukesh Desai, Manisha Madkaikar

**Affiliations:** ^1^Department of Pediatric Immunology and Leukocyte Biology, Indian Council of Medical Research-National Institute of Immunohaematology, Mumbai, India; ^2^Department of Immunology, Bai Jerbai Wadia Hospital for Children, Mumbai, India; ^3^Department of Bone Marrow Transplant, Bai Jerbai Wadia Hospital for Children, Mumbai, India; ^4^Department of Pediatric Hematology, Oncology, Blood and Marrow Transplantation, Apollo Hospitals, Chennai, India; ^5^Comprehensive Thalassemia Care, Pediatric Hematology-Oncology & Bone Marrow Transplantation Centre, Mumbai, India; ^6^Department of Pediatric Hemato-Oncology, Kanchi Kamakoti CHILDS Trust Hospital, Chennai, India; ^7^Medgenome Labs Pvt Ltd., Narayana Health City, Bommasandra, India; ^8^Department of Pediatrics, Post Graduate Institute of Medical Education and Research, Chandigarh, India; ^9^Department of Clinical Immunology and Rheumatology, Sanjay Gandhi Postgraduate Institute of Medical Sciences, Lucknow, India; ^10^Department of Pediatrics, All India Institute of Medical Sciences, Nagpur, India; ^11^KAHER'S Jawaharlal Nehru Medical College, Belagavi, India; ^12^Department of Pediatrics, Sri Ramchandra Institute of Higher Education and Research, Chennai, India; ^13^Department of Pediatrics, All India Institute of Medical Sciences, Bhopal, India; ^14^P.D. Hinduja Hospital, Mumbai, India; ^15^Department of Haematology, Seth G. S. Medical College and King Edward Memorial Hospital, Mumbai, India; ^16^Lilavati Hospital and Research Centre, Mumbai, India; ^17^Department of Pediatric Oncology, Tata Memorial Hospital, Mumbai, India; ^18^Kokilaben Dhirubai Ambani Hospital, Mumbai, India; ^19^Kashyap Nursing Home, Mumbai, India

**Keywords:** familial hemophagocytic lymphohistocytosis, perforin, degranulation, HLH-targeted therapy, flow cytomertry, NGS

## Abstract

Hemophagocytic lymphohistiocytosis (HLH) is a syndrome of immune dysregulation characterized by hyperactivation of the immune system, excessive cytokine secretion and severe systemic inflammation. HLH is classified as familial (FHL) when associated with mutations in *PRF1, UNC13D, STX11*, and *STXBP2* genes. There is limited information available about the clinical and mutational spectrum of FHL patients in Indian population. This study is a retrospective analysis of 101 molecularly characterized FHL patients over the last 10 years from 20 different referral centers in India. FHL2 and FHL3 together accounted for 84% of cases of FHL in our cohort. Patients belonging to different FHL subtypes were indistinguishable based on clinical and biochemical parameters. However, flow cytometry-based assays viz. perforin expression and degranulation assay were found to be specific and sensitive in diagnosis and classification of FHL patients. Molecular characterization of respective genes revealed 76 different disease-causing mutations including 39 (51%) novel mutations in *PRF1, UNC13D, STX11*, and *STXBP2* genes. Overall, survival was poor (28%) irrespective of the age of onset or the type of mutation in our cohort. Altogether, this article sheds light on the current scenario of FHL in India. Our data reveal a wide genetic heterogeneity of FHL in the Indian population and confirms the poor prognosis of FHL. This study also emphasizes that though mutational analysis is important for diagnostic confirmation of FHL, flow cytometry based assays help significantly in rapid diagnosis and functional validation of novel variants identified.

## Introduction

Familial hemophagocytic lymphohistiocytosis (FHL) is a disorder of immune dysregulation characterized by persistent high-grade fever, progressive cytopenias, hepatosplenomegaly and systemic inflammation. It is an autosomal recessive disorder and affects mostly infants and young children, but has also been reported in adolescents and adults (1, 2). So far, based on the gene mutations observed, FHL is categorized as FHL2 (*PRF1)*, FHL3 (*UNC13D)*, FHL4 (*STX11)*, and FHL5 (*STXBP2)* encoding for Perforin, Munc13-4, Syntaxin11, and Syntaxin binding protein 2, respectively (3). These proteins play a fundamental role in lymphocyte cytotoxicity. FHL1 (9q21.3-22) was identified by homozygosity mapping of four inbred families of Pakistani origin (4); though, the disease-causing gene in this locus is yet been unknown.

As the clinical features are similar to those with various infections and inflammatory disorders, diagnosis can often be missed. Though, Histiocyte Society has established HLH diagnosis criteria, which includes clinical manifestations and laboratory findings; it does not help in classifying FHL patients and identifying the underline genetic defect.

Impaired function of NK cells and cytotoxic T lymphocytes (CTLs) due to inherited defect in the granule mediated cytotoxicity is the hallmark of FHL patients (5). Thus, for the diagnosis of these patients, evaluating the function of NK cell and CTLs is crucial. In recent years, assays based on flow cytometry have been developed for evaluating NK cell and CTL cell functions viz. measurement of intracellular perforin expression and degranulation assay determined by upregulation of CD107a expression (6, 7). Granule release assay (GRA) is a screening test for detection of FHL3, FHL4, and FHL5 patients. Measurement of intracellular perforin levels serves as a phenotypic assay in identifying FHL2 patients. These tests serve as rapid screening tests for identifying FHL patients. However, molecular characterization of the respective genes is essential for the final diagnostic confirmation. Identifying underlying genetic defect is also important for offering genetic counseling and prenatal diagnosis in affected families.

The incidence of the four FHL subtypes varies significantly in different ethnic groups; also certain mutations are unique or commonly seen in a particular population (1, 8–10). Understanding this pattern of mutation in a particular population not only helps in cost-effectively designing strategies for mutation screening but may also have epidemiological implications. However, very limited data is available on FHL from India. Thus, in this retrospective study, we report one of the largest series on general clinical features; immunological and molecular findings and outcome of FHL in 101 patients from 20 different referral centers of India over the last 10 years.

## Materials and Methods

### Enrollment of Patients and Ethics Statement

This study was approved by Institutional Ethics committee (IEC) for Human subjects of ICMR-National Institute of Immunohematology (NIIH). Ethical clearance was obtained at each center and patients received research information and provided written informed consent to participate in this study. Patients fitting into HLH criteria of the Histiocyte Society (5) referred to ICMR-NIIH or collaborating FPID centers or other tertiary care centers in India from 2010 to 2020 were included in this study. Detailed clinical and family history was recorded for these patients. All the procedures involving human subjects were performed in accordance with the international ethical standards.

### Diagnosis of HLH

As a part of diagnostic workup of HLH, perforin expression and degranulation assay on NK cells were performed as previously described (6, 7, 11). Lymphocyte subset analysis of patient samples was performed using Multitest 6-color TBNK reagent (Becton Dickinson).

Further, confirmation of molecular diagnosis was done either by direct Sanger sequencing (in perforin deficient patients) or by targeted Next-Generation sequencing (NGS) or clinical whole-exome sequencing (WES) (in patients with abnormal degranulation).

### Bioinformatics and Statistical Analysis

The sequences obtained were compared with the reported gene structures (*PRF1, UNC13D, STX11, STXBP2*: NCBI) using the BLASTN program (http://www.ncbi.nlm.nih.gov/BLAST). Nature of any novel sequence variant was analyzed by using different prediction software i.e., PolyPhen-2 (http://genetics.bwh.harvard.edu/pph2/), SIFT (http://www.sift.jcvi.org/). For splice region variants, Human Splicing Finder tool is used (https://hsf.genomnis.com/).

Data was presented in terms of median and percentages. For a comparison of >2 groups, One-way analysis of variance (ANOVA) test was used. Mann-Whitney U-test was used for comparing groups with non-parametric data. The *p*-values ≤0.05 were considered statistically significant. GraphPad Prism (Chicago, IL, USA) version 5 was used for statistical calculations.

## Results

### The Pattern of Familial HLH

In this study, we have included 101 molecularly characterized FHL patients from the year 2010–2020. Of these, 50 patients were identified as FHL2, 35 were FHL3, seven were FHL4 and nine were FHL5 based on the defective genes. Thus, in our Indian cohort, perforin deficiency was found to be the commonest (49%) followed by Munc13-4 (35%) deficiency.

### Patient Characteristics

The baseline characteristics of these patients are detailed in Table 1, Supplementary Table 1, which also defines the patients according to their genotype. Of these patients, 67% of patients were male, with a male: female ratio of 2:1. The median age of diagnosis for the entire cohort was 12 months (range: 8 days−33 years) with around 55% of the patients presenting within 1 year of age. Consanguinity was observed in 43% (37/86) patients and 28% (22/80) patients had a family history of an affected sibling. No significant difference in age of presentation was observed amongst the FHL groups.

**Table 1:** Comparison of patient characteristics in different groups of FHL patients.

**Genotype**	**FHL2**	**FHL3**	**FHL4**	**FHL5**
**Characteristics**				
Number of patients	50	35	7	9
Male	31 (62%)	23 (64%)	6 (86%)	8 (89%)
Family history	12/37 (32%)	8/32 (27%)	1/6 (17%)	1/5 (20%)
Consanguinity	16/40 (40%)	15/32 (48%)	2/7 (28.5%)	4/7 (57%)
**Age of diagnosis**				
<3 months	12 (24%)	10 (28%)	0 (0%)	1 (11%)
3–6 months	13 (26%)	6 (19%)	1 (14%)	2 (22%)
6–12 months	4 (8%)	5 (14%)	1 (14%)	1 (11%)
1–4 years	7 (14%)	9 (25%)	2 (29%)	0 (0%)
>4 years	14 (28%)	5 (14%)	3 (43%)	5 (56%)
Median age of presentation (range) (in months)	6 (0.25–384)	6 (1–396)	18 (3–144)	48 (2–168)
Fever	46/50 (92%)	31/33 (94%)	7/7 (100%)	7/7 (100%)
Hepatosplenomegaly	45/46 (98%)	30/32 (94%)	7/7 (100%)	9/9 (100%)
Bicytopenia	29/40 (72.5%)	16/23 (69.5%)	6/6 (100%)	7/9 (78%)
Hyperferritinemia	39/41 (95%)	25/28 (89%)	7/7 (100%)	6/7 (86%)
Elevated sCD25 levels*	19/19 (100%)	5/6 (83%)	2/2 (100%)	ND
Hemophagocytosis	38/40 (95%)	18/25 (72%)	6/6 (100%)	5/5 (100%)
Hypertriglyceridemia	30/39 (77%)	23/27 (85%)	6/6 (100%)	7/7 (100%)
Hypofibrinogenemia	21/33 (64%)	18/22 (82%)	3/4 (75%)	2/5 (40%)
CNS symptoms	9/32 (28%)	9/20 (45%)	2/6 (33%)	1/3 (33%)

* *sCD25 levels compared to age-matched healthy controls*.

In our cohort, trigger for FHL development could be identified in 18 patients. Viral infection was found to be the most common trigger in nine patients while bacterial infections are associated with FHL in four patients. Parasitic and fungal infection was seen in one patient each. Three FHL patients had hematological malignancy. However, in rest of the patients, information of triggers for FHL development was not available. 74% of patients fulfilled at least 5/8 HLH diagnostic criteria; whereas 22% of patients fulfilled 4/8 criteria. A pre-symptomatic diagnosis in view of strong family history was achieved in 4% patients. The common clinical presentations seen in all groups of FHL were fever (94%) and hepatosplenomegaly (97%). Hyperferritinemia was observed in 93% cases while 88% patients presented with hemophagocytosis on bone marrow examination. 83.5 and 69% of the patients had hypertriglyceridemia and hypofibrinogenemia, respectively. Deranged liver enzymes were observed in 69% patients. CNS manifestations were seen in 32% patients. Seizure disorder was the commonest CNS manifestation while cerebral palsy and peripheral neuropathy was observed in one patient each. Erythematous skin rash was observed in 19% patients. No significant difference in clinical features was observed between the different subtypes of FHL patients (Table 1).

All FHL patients, except six patients (two FHL2, three FHL3, and one FHL5), had serum ferritin levels >500 ng/ml. Both the FHL2 patients had a family history and they were asymptomatic when referred to our laboratory. Three FHL3 patients (P58, P73, and P74) with <500 ng/ml ferritin had borderline ferritin levels (471, 474, and 488 ng/ml, respectively). Overall, 65% FHL patients had ferritin levels between 500 and 10,000 ng/ml while 17% of FHL2 and 11% of FHL3 patients had ferritin levels >20,000 ng/ml. Ferritin levels did not differ amongst the FHL subtypes. sCD25 levels were available in 27 patients and were elevated in 96% patients.

## Immunological Findings

### Lymphocyte Subset Analysis

Lymphocyte subset analysis (enumerating T cells, Tc cells, Th cells, NK cells, and B cells) was performed in 37 patients (18 FHL2, 11 FHL3, 2 FHL4, and 6 FHL5). Though lymphopenia was observed in 49% of the patients, the frequency and absolute counts of lymphocyte subsets did not differ amongst the subsets of FHL (Supplementary Figure 1).

### Perforin Expression

**Figure 1:**
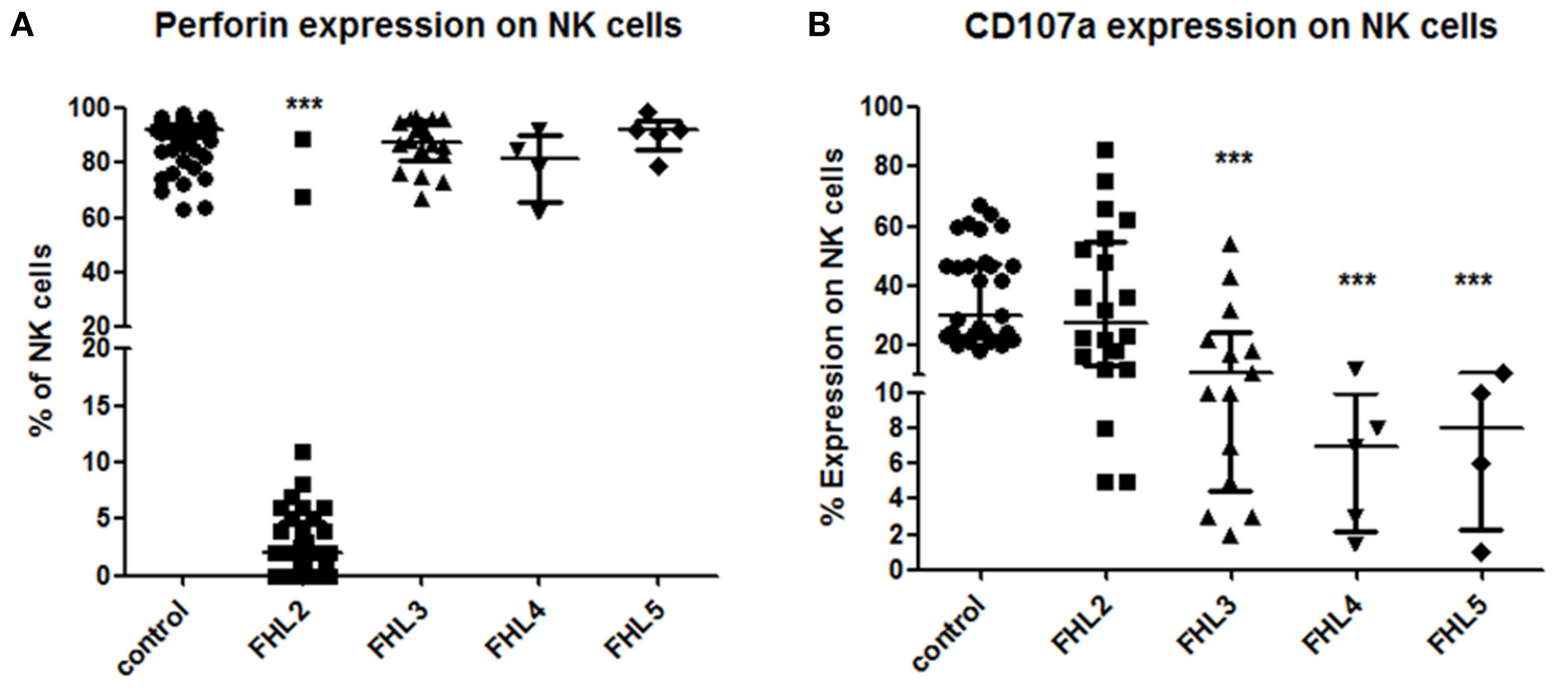
Comparison of Perforin expression and CD107a expression on stimulated NK cells in different groups of FHL patients **(A)** Perforin expression **(B)** CD107a expression. One way ANOVA test was used to evaluate differences between activation markers in different groups. ^***^P < 0.001.

Perforin expression could be checked by flow cytometry in 40 out of 50 FHL2 and 30 out of 51 FHL3/4/5 patients. 38/40 FHL2 (95%) had significantly lower expression of perforin on NK cells (median 2%; reference range 72 ± 2%) than the healthy controls (median 92%) and FHL patients in other groups (median 88%) (*p* < 0.001) (Figure 1A). 92.5% of the FHL2 patients had ≤10% perforin expression on NK cells with 62.5% having ≤2% expression on NK cells. The cut-off of <10% perforin expression on NK cells to identify patients with biallelic mutations compared to patients with normal sequencing results yielded a sensitivity of 92.5% the specificity of 100%, the positive predictive value of 100%, and negative predictive value of 93.75% with an accuracy of 97% (AUC of 0.9852) (Supplementary Figure 2A).

### Degranulation Assay

CD107a degranulation assay could be performed in 20 out of 50 FHL2, 18 out of 35 FHL3, five out of seven FHL4 and four out of nine FHL5 patients. Defective degranulation was defined as ≤10% CD107a expression on stimulated NK cells. All FHL4 and FHL5 (100%) and 12/18 FHL3 patients (79%) patients had defective degranulation assay (Figure 1B). Three FHL3 patients (P56, P59, and P61) had borderline CD107a expression on NK cells (22, 18, and 17%, respectively), while three FHL3 patients (P62, P63, and P69) had degranulation within a normal range (Median 37.5%; 22–54%; reference range 28 ± 8%). Additionally, three FHL2 patients (P27, P36, and P39) had defective degranulation on NK cells (Figure 1B). The cut-off of <10% CD107a expression on NK cells for diagnosis of FHL patients with degranulation defect yielded a sensitivity of 73.91%, the specificity of 88.24%, the positive predictive value of 89%, and negative predictive value of 81% with an accuracy of 85% (AUC, 0.8939) (Supplementary Figure 2B).

### Mutation Spectrum

Out of 102 molecularly characterized FHL patients, details of the mutation spectrum were available in 88 FHL patients (44 FHL2, 29 FHL3, six FHL4, and nine FHL5). This manuscript involves retrospective data from multiple centers all over India of last 10years. Thus, retrieving data of exact annotation of mutations of very old patients (six FHL2, seven FHL3, and one FHL4) was difficult. However, on records, these patients have proven mutations with details of underlying defective gene mentioned. Also for these patients' transplant details and outcome could be traced and hence are included in this study.

Total of 76 different disease-causing mutations were identified in four HLH-related genes (Table 2). Of these 76 mutations identified; 42 (55%) were missense mutations, 7 (9%) were nonsense mutations, 18 (24%) were frameshift mutations and 9 (12%) were intronic and splice site mutations (Table 2). 63 (72%) patients were detected to have homozygous mutations, 12 (14%) had compound heterozygous variants and 12 (14%) had a single heterozygous mutation (Figure 3A). In total, 39 (51%) novel mutations were identified that included 15 missense mutations, four nonsense mutations, 16 frameshift mutations and four splice site and intronic mutation.

**Table 2:** Different mutations identified in patients with FHL.

	**FHL2 (*n* = 44)**	**FHL3 (*n* = 28)**	**FHL4 (*n* = 6)**	**FHL5 (*n* = 9)**
No of mutations	33	30	5	8
Novel mutations	17 (51%)	18 (60%)	2 (40%)	2 (25%)
**Type of mutation**				
Missense	24 (73%)	11 (37%)	3 (60%)	5 (62%)
Nonsense	5 (15%)	1 (3%)	1 (20%)	0 (0%)
Frameshift	4 (12%)	12 (40%)	1 (20%)	0 (%)
Splice-site and intronic	0 (0%)	6 (20%)	0 (0%)	3 (38%)

In the 44 FHL2 patients, we identified 33 different *PRF1* mutations with a total of 17 novel variants (Table 2). Homozygous mutations were found in 62% of FHL2 patients. The most frequent mutations were c.386G>C (p.W129S) which was present in five patients; c.1349C>T (p.T450M) which was present in five patients; c.528_529delinsAA (p. C176X) which was present in three patients; and c.673C>T (p.R225W) which was also seen in three patients. Overall, missense mutations were commonly found in FHL2 patients (48%) in our cohort.

In the 28 patients with FHL3, 30 different mutations with 18 novel variants were identified (Table 2). Homozygous mutations were seen 62% of FHL3 patients. Frameshift, intronic and splice site mutations were more frequently observed in our FHL3 patients (59%). The most frequent mutations were c.762delC (p.C255AfsX73) (frameshift deletion); and c.1822del (V608CfsX16) (frameshift deletion); and c.858+1G>A (intronic splice site mutation) which were present in two patients, respectively.

Also, in the six FHL4 and nine FHL5 patients, we identified five and eight different mutations, respectively (Table 2). c.173T>C (L58P) was found in two FHL4 patients. Homozygous intronic splice site variant 1247_1G>C in STXBP2 gene was observed in three FHL5 patients.

### Genotype-Phenotype Correlation

**Figure 2:**
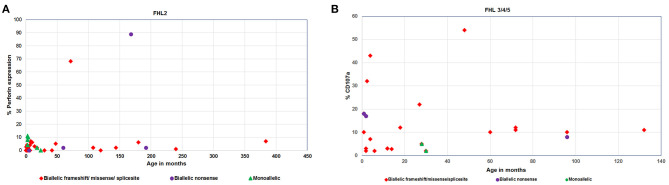
**(A)** Perforin expression and **(B)** CD107a degranulation in FHL patients with different age categories and genetic background.

**Figure 3:**
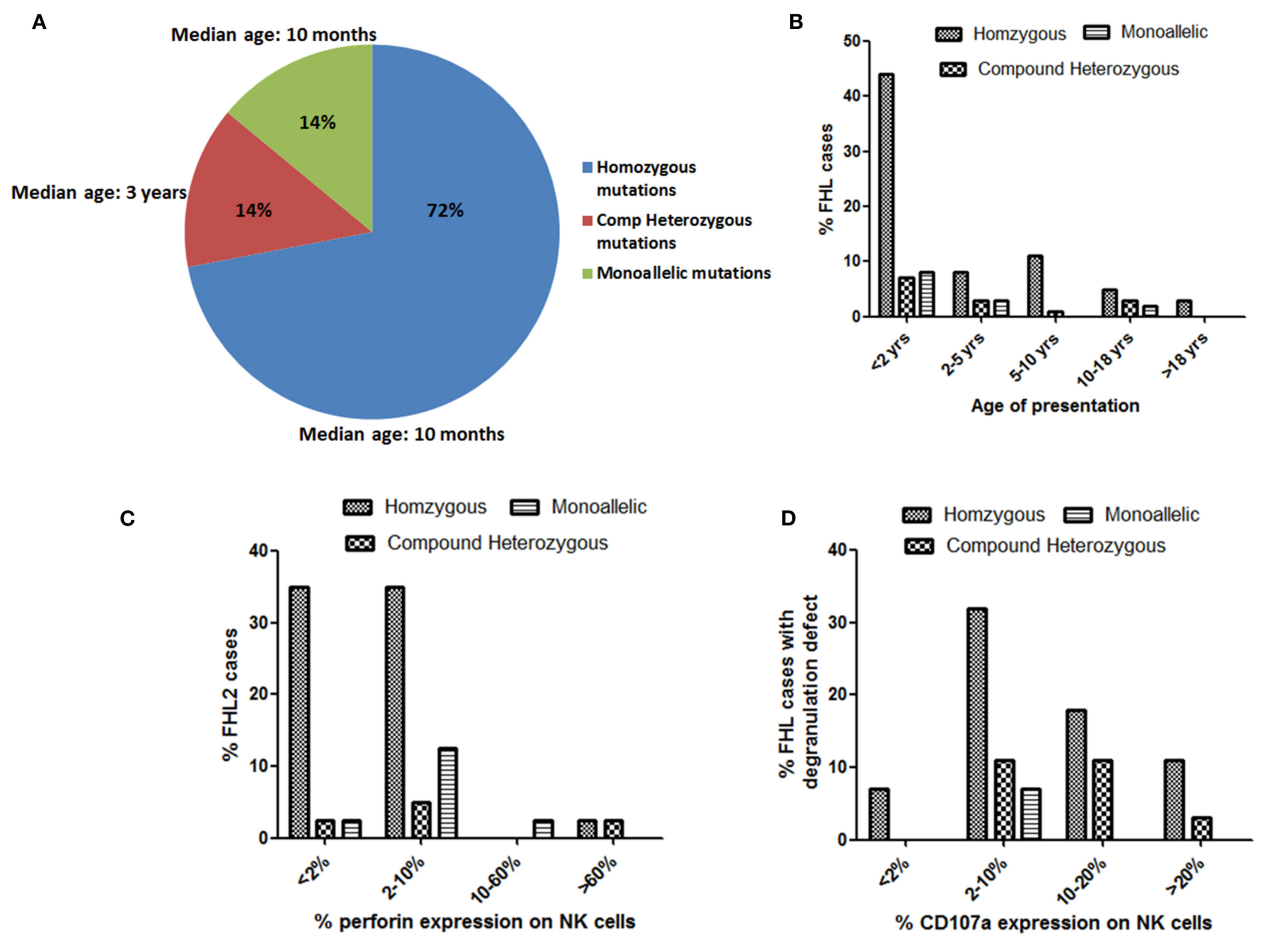
Evaluation of age of presentation of disease **(A,B)**, Perforin expression **(C)** and CD107a expression **(D)** on stimulated NK cells in different groups of FHL patients with associated genetic profiles.

Irrespective of FHL subtype, 53% patients harboring homozygous mutations presented below 1 year of age (median age of presentation was 10 months) while patients with compound heterozygous mutations had later onset of disease (median age of presentation was 3 years). Similarly, 12 patients with a monoallelic mutation in FHL genes had a median age of presentation of 10 months (Figures 2, 3A,B).

Irrespective of the genetic background i.e., presence of homozygous/compound heterozygous/monoallelic mutations, FHL2 patients had perforin expression ≤10% except for patient P34 (with homozygous mutation) and patient P41 (with compound heterozygous mutation). P34 and P41 had 68 and 89% perforin expression on NK cells, respectively (Figures 2A, 3C). However, in P41, mean fluorescence intensity (MFI) of perforin expression was very low (1.83) as compared to healthy control (19.83); indicating that not only expression but MFI of staining also has to be considered during interpretation of the results.

From 43 patients of FHL3/4/5 for whom mutation details were available; 32 patients had homozygous mutations in the corresponding genes (Figures 2B, 3D). Seven patients of FHL3/4/5 had compound heterozygous mutations in the respective genes while the remaining four patients had a monoallelic mutation in the respective genes. Five out of these seven patients with compound heterozygous variants had CD107a degranulation on NK cells ≤10%, one patient P61 had borderline degranulation and one patient P69 had degranulation in the normal range. Additionally, two FHL3 patients (P62 and P63) with homozygous frameshift mutations had normal degranulation assay. Amongst the four patients with monoallelic mutations, CD107a degranulation could be performed in two patients and it was found to be abnormal.

Although majority of the patients had typical HLH manifestations, four FHL patients (three FHL2 i.e., P6, P7, P14, and one FHL5 i.e., P93) are identified with atypical clinical presentations like lymphoma, leukemia, or autoimmune diseases. All of these patients received treatment for the respective pathological conditions and then were diagnosed with HLH. As there was a delay in diagnosis of FHL due to the unusual clinical presentations, all these patients succumbed to the disease.

### Treatment and Outcome

Of the 101 cases of FHL, 13 patients were lost to follow up and the outcome and remission status could be reported in 88 patients. Seven patients expired before starting the HLH treatment, while 51 patients expired even after receiving HLH-2004/94 protocol. Of the latter, 43 patients did not achieve remission and expired; another eight showed a relapse of the disease post-remission and could not be salvaged. Hematopoietic cell transplantation (HCT) being the definitive treatment, 18 patients underwent BMT. Thirteen are currently doing well while five patients expired due to post-transplant complications. Twelve patients are currently in remission and awaiting transplantation. The treatment and outcome of HLH patients in our study are summarized in Table 3.

**Table 3:** Outcome of therapy seen in different FHL groups.

	**FHL2** ***n* = 50**	**FHL3** ***n* = 35**	**FHL4** ***n* = 7**	**FHL5** ***n* = 9**	**Total** ***n* = 101-**
Expired before receiving the protocol	6	0	0	1	7
Expired even after receiving the protocol	28	16	3	4	51
In remission while on protocol and awaiting transplantation	3	5	2	2	12
Well post-HCT	4	7	1	1	13
Expired post-HCT	3	2	0	0	5
Lost to follow-up	6	5	1	1	13

## Discussion

FHL is a genetically heterogeneous disorder of immune dysregulation affecting the cytotoxic function of lymphocytes (3, 5). The pattern of FHL has been reported to vary significantly in different ethnic groups (8–10, 12), however, its prevalence in Indian population is not known. In this study, we have comprehensively evaluated clinical, immunological and molecular findings in 101 molecularly characterized FHL patients from 20 different centers of India. To the best of our knowledge, this is one of the largest studies on the spectrum of FHL in Indian population. This study also evaluates the utility of flow cytometry based assays for efficient diagnosis of FHL patients.

In accordance with previous studies (1, 3, 9, 10, 13), 84% of our FHL patients were either FHL2 or FHL3 (50 and 35%, respectively); while the remaining 16% were FHL4 and FHL5 patients. Also as reported in various retrospective studies in other populations, in our cohort as well prolonged fever, hepatosplenomegaly, hemophagocytosis and hyperferritinemia were the most common clinical presentations (3, 9, 12, 14). CNS manifestations were more common in our FHL3 patients, which is consistent with that reported in literature (15, 16). Although FHL has widely been considered as a disease of infancy (3, 10, 14), 43% of our FHL patients had onset beyond 1year of age, with 24% of FHL2 patients presenting beyond 4 years of age and had severe disease. This suggests the importance of screening for underlying genetic defect in all HLH patients irrespective of their age.

As reported in previous literature (5, 12, 14), 22% of the patients in our cohort did not fulfill the Histiocyte society criteria (5) suggesting certain limitations of existing diagnostic criteria. Many patients may not meet these criteria early during the disease, and some patients may never meet criteria including those with atypical clinical presentations like isolated central nervous system disease (17–19). In some patients, information on levels of sCD25 or NK-cell cytotoxicity may not be available. So in such scenario, measurement of additional screening markers like flow cytometric detection of perforin, CD107a degranulation, T cell upregulation of HLA-DR, serum levels of CXCL9, IL18 are gaining attention for HLH diagnosis and differentiating HLH patients from patients with rheumatological diseases (20).

Nowadays, molecular analysis using targeted NGS or clinical WES is the preferred mode of FHL diagnosis (21). But such molecular diagnosis can be challenging in terms of cost, genetic heterogeneity, analytic difficulty, turnaround time and availability. Thus, in this scenario performing functional screening of cytotoxic cells by flow cytometry is complementary, as the results are available within 72 h. Currently, there are only two Centers of Excellence (COE) in India which provide Immunoassays like flow cytometry based detection of perforin expression and CD107a degranulation on NK cells. Immunoassays (at least one) could be performed in 70 patients in our cohort. In majority of the cases, results are available within 48 h except for the few traveled samples where additional time is required for transport. There are several peripheral centers which send the samples simultaneously for phenotypic and molecular analysis. In recent years, assays based on flow cytometry for evaluating NK cell and CTL cell functions are developed (6, 7, 22–24). In addition to cytotoxicity assays, quantification of perforin, SAP and XIAP proteins which are involved in granule-mediated exocytosis are useful in the diagnosis of primary HLH patients (22, 25–27). Along with NK cells, evaluation of CD107a degranulation on cytotoxic T lymphocytes (CTL) is recently reported in the diagnosis of FHL (28, 29). In our cohort, perforin expression and degranulation assay both had high specificity and sensitivity for distinguishing patients with HLH-associated mutations, as seen in previous studies (11, 23, 24).

Molecular characterization of patients in our cohort, revealed 39 novel variants (51%), highlighting the diverse molecular findings in Indian population compared to other populations reported in the literature (1, 12, 30, 31). The mutations identified in HLH-related genes viz. *PRF1, UNC13D, STX11*, and *STXBP2* genes were widely spread across the coding regions of the respective genes and no founder mutation could be identified in our population. As consanguinity is mainly seen (43%) in our Indian cohort, homozygous mutations in FHL genes are majorly found in the study.

The minimal or complete absence of perforin protein is commonly associated with the most detrimental *PRF1* gene mutations while, compound heterozygous *PRF1* gene missense mutations may encode partially active perforin and are predominantly identified in older patients with milder clinical manifestations (1, 32, 33). In our cohort, most patients (82.5%) harboring homozygous mutation had perforin expression <10% (Figures 3B,C). In two patients viz. P34 and P41, perforin expression was found to be 68 and 89%, respectively, and had later onset of disease. Patient P34 had c.136G>A (p.E46K) which is a reported missense mutation. This mutation leads to a defect in the MACPF domain of perforin protein which consists of ~349 amino acids stretching in the middle of the protein and is thought to be involved in pore formation. In P41, though perforin expression was 89%, MFI of staining was low. This patient carried compound heterozygous mutation including missense mutation (c.1349 C>T; p.T450M) and nonsense mutation (c.1519G>T; p.E507X) affecting the C2 domain of perforin protein which is involved in Ca^2+^ and membrane binding. Both these patients had late onset of the disease (6 and 14 years, respectively) probably attributing to residual perforin expression. Despite the presence of perforin expression on NK cells and late-onset of disease, these patients presented with severe clinical manifestations. Mutations identified in these patients might be affecting the function or structure of perforin protein but not its expression, thus highlighting the fact that presence of a protein does not rule out its functional or structural defect. So, in case of high clinical suspicion, molecular analysis along with functional assays should be considered.

All FHL2 patients had normal degranulation pattern except patient P27, P36, and P39. P27 was only 45 days old and abnormal degranulation pattern could be attributed to the immature immune system at this early age (34). Unfortunately, degranulation assay could not be repeated in any of these patients. Majority of the patients with a homozygous mutation in either *UNC13D* or *STX11* or *STXBP2* had either defective (≤10%) degranulation on stimulated NK cells (Figure 3D). Borderline NK degranulation (22, 18, 10, and 17%) was observed in patients 56, 59, 60, and 61, respectively. Three of these patients (56, 59, and 61) harbored novel frame-shift mutation in homozygous state in *UNC13D* gene leading to premature stop codon causing truncated protein formation while patient 60 harbored compound heterozygous missense mutation (one novel and one reported mutation) in *UNC13D* gene. Although these patients had degranulation ≥10%, it was lower than the healthy controls (22–54%). Thus, indicating that these mutations definitely had effect on the NK cell degranulation pattern. Amongst the FHL3 patients, three patients (62, 63, and 69) harboring novel frameshift mutations in *UNC13D* gene had NK cell degranulation within normal range. NK cell degranulation assay though has high sensitivity and specificity, it is a screening test and hence can have false negative results. Similar findings were observed in study by Bryceson et al. (11) wherein degranulation assay was evaluated for diagnosis of FHL and was found that 4% of the patients with genetically determined degranulation defects had NK cell degranulation >10% while 7% had above 20% (11). Thus, indicating that, in case of high suspension or family history, one has to go ahead with molecular analysis irrespective of the outcome of functional assays. Evaluation of parents' status in these patients and *in vitro* functional assays in future may help in assigning pathogenicity in such novel variants.

Interestingly, in our cohort 12 patients (eight FHL2, four FHL3) though harbored monoallelic mutations, had severe clinical manifestations. Phenotypic assays could be performed in nine patients and were abnormal in eight of them. Clinical follow up was available in seven patients. Six of them expired while one required HCT because of relapse and currently well. Of these patients, mutations in three patients (P76–P78) were identified by Whole exome sequencing thus ruling out the possibility of compound heterozygous mutations in these patients. Presence of monoallelic mutations has also been observed in previous studies and there is a growing evidence that monoallelic variants can also contribute to FHL (16). Though involved in the pathogenesis of the disease, monoallelic variants may not be sufficient to initiate the disease phenotype alone. Additional unidentified genetic defects or possibly even environmental factors may contribute to the development of HLH (2, 35). The digenic mode of inheritance (35) and deep intronic variants [c.118-308C>T, c.118-307G>A and 253-kb inversion] in UNC13D gene (36, 37) are described in FHL patients carrying monoallelic variants support this inference. The simple possible explanation is that the variants like promoter mutation, deep intronic variants and big deletions can be missed with the commonly used molecular techniques. Another possibility is that some patients (especially with degranulation defects) might possess pathogenic variants in additional genes that contribute to the development of HLH (35, 38). On the other hand, few monoallelic mutations may confer dominant-negative function to the encoded protein interfering with the cytotoxic function of lymphocytes although the exact mechanism and clinical relevance of these monoallelic mutations need to be explored (39).

Since cellular cytotoxicity plays a crucial role in immune surveillance and tolerance, it gives rise to an association of FHL with cancer, autoimmune susceptibility and other manifestations (16). In our cohort, we identified four FHL patients with atypical initial presentations, namely, two FHL2 patients with malignancy (P6 and P7), one FHL5 patient with ALPS (P93), and one FHL2 patient with isolated neurological relapse (P14). Particularly, isolated CNS manifestations in FHL can be challenging to diagnose (17, 40, 41). These findings highlight some of the atypical manifestations in FHL which may delay diagnosis in selected cases.

Management of HLH is mainly focused on treating the infectious trigger, suppression of hyperactive immune system and correction of an underlying genetic defect. Prompt initiation of immunosuppressive chemotherapy is essential for improved outcome. This can be achieved by increasing the awareness about the availability of immunoassays for diagnosis of HLH helping in early initiation of therapy and preparation for HCT. The underlying triggering infections must be extensively evaluated as the disease activity can be improved by managing the etiology. Some biomarkers like sCD25 levels and sCD163 levels are available at only in limited centers. Awareness to incorporate these biomarkers along with the fall in ferritin levels must be used actively in the management of these patients. In cases of refractory HLH, other novel therapies like Emapalumab or Alemtuzumab are recommended but they are currently not easily available in India (20, 42). In our study cohort, 63 patients expired either before initiating therapy or when on therapy or post-HCT. Thirteen patients are well post-HCT while twelve patients are in remission on HLH protocol and awaiting bone marrow transplantation. Two FHL2 patients having late onset of disease, relapsed when treatment was tapered and hence, emphasizing on the fact that even patients with adult-onset FHL need HCT for better outcome (13). Thus, though early initiation of treatment is necessary for the survival of HLH patients; HCT is the only curative therapy for patients with a known genetic defect and also for patients with recurrence/relapse of the disease despite adequate therapy (though the genetic cause is not known). HLA-matched sibling donor is the preferred donor for FHL patients due to the minimized risk of GVHD. However, various reports on variation in the age at onset within each family, case studies reporting the adult FHL patients and also symptomatic patients harboring monoallelic mutations add to the dilemma of choosing a donor for HCT (43, 44). The studies from our lab have shown that parents and siblings of few FHL2 patients were asymptomatic carriers for the respective mutations in a heterozygous state and had partial (<50%) perforin expression (unpublished data) as has been reported previously (16). Thus, in such cases, the decision of whether to go ahead with HCT from these potential donors is a dilemma especially, in a country like India where transplantation procedures are often not feasible either due to unavailability of HLA-matched donor or the unaffordable cost of therapy.

Thus, to summarize, this is one of the largest studies of retrospective data on the clinical, immunological and molecular spectrum of FHL patients in Indian population. Availability of flow cytometry based assays and NGS have significantly improved the diagnosis and management of FHL patients. Both flow cytometry based assays and molecular studies are complementary to each other and aid in key management decisions. Forthcoming advances in FHL syndrome recognition to minimize delays in diagnosis and modified treatment and transplant approaches will continue to improve patient outcomes.

## Data Availability Statement

The raw data supporting the conclusions of this article will be made available by the authors, without undue reservation.

## Ethics Statement

The studies involving human participants were reviewed and approved by Institutional Ethics Committee (IEC) for human subjects of ICMR-National Institute of Immunohematology (NIIH). Written informed consent to participate in this study was provided by the participants' legal guardian/next of kin.

## Author Contributions

SS and MKe analyzed the data and wrote the manuscript. RM and UB wrote the manuscript and obtained clinical details. MGu, ADa, JA, MKu, SSh, GH, PS, NJ, and NN performed the laboratory investigations. RV, SS-D, PKa, ADh, and PG involved the molecular investigations. PT involved in collection of samples and maintaining clinical details. VG, AP, RR, RU, RS, PKi, MS, DM, PV, AB, AR, AA, UP, MGi, AC, AS, DJ, NC, NS, FJ, SC, SK, BA, SSe, and ML supervised the clinical care and management of patients. MD and MM supervised the study and reviewed the manuscript. All authors contributed to the article and approved the submitted version.

## Conflict of Interest

RV was employed by the company Medgenome, Pvt. Labs, Bangalore, India. The remaining authors declare that the research was conducted in the absence of any commercial or financial relationships that could be construed as a potential conflict of interest.
