# Supplementary material for: The Spectrum of Clinical, Immunological, and Molecular Findings in Familial Hemophagocytic Lymphohistiocytosis: Experience From India
Source: Front Immunol. 2021 Mar 5;12:612583. doi: 10.3389/fimmu.2021.612583 (PMC7973116; doi:10.3389/fimmu.2021.612583)
Supplement: Supplementary file 1 [file Data_Sheet_1.doc]

**The spectrum of clinical, immunological and molecular findings in Familial hemophagocytic lymphohistiocytosis (FHL): Experience from India**

**Supplementary Figures:**


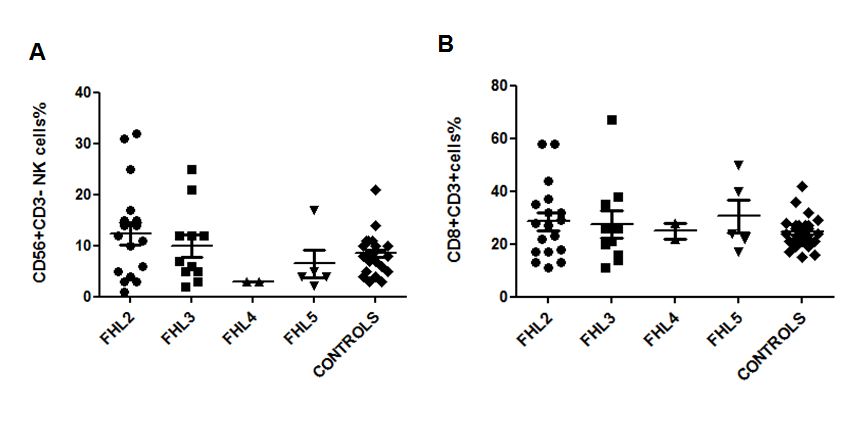


**Supplementary Figure 1:** The percentage of NK cells (**A**) and CD8 cells (**B**) in FHL patients along with healthy controls as evident from lymphocyte subset analysis


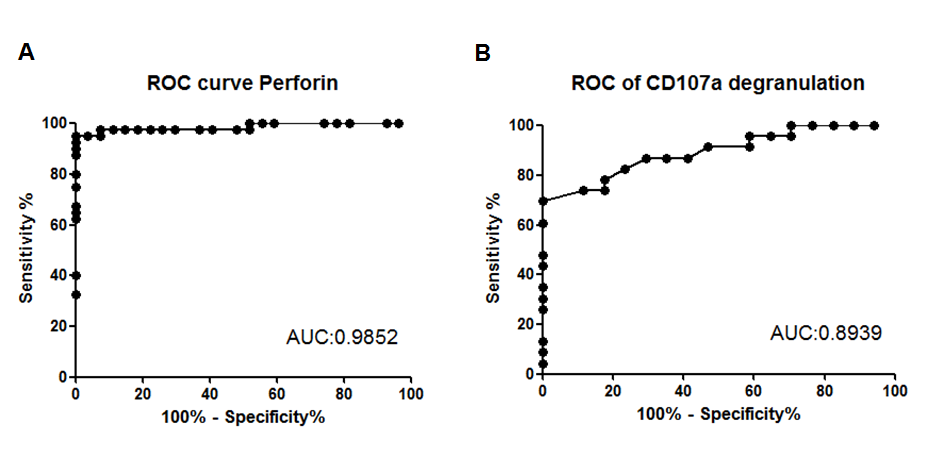


**Supplementary Figure 2:** **A** ROC analysis of perforin expression on NK cells to detect FHL2 patients from FHL3/4/5 patients using GraphPad Prism 5 **B.** CD107a degranulation on NK cells to detect FHL patients with degranulation defects from FHL2 patients

**Supplementary tables**

**Supplementary table 1: Characteristics of FHL patients**

| **No** | **Sex** | **Age at diagnosis (months)** | **Mutation** | **Protein change** | **Perforin expression on NK cells %** | **NK cell degranulation%** | **Outcome** |
| --- | --- | --- | --- | --- | --- | --- | --- |
| **FHL2** |  |  | ***PRF1*** |  |  |  |  |
| 1* | F | 3 | 833C>A | A278D | 8 | NA | expired |
| 2 | F | 2 | 386G>C | W129S | 4 | 18 | expired |
| 3 | F | 5 | 490C>T | Q164X | 0 | NA | expired |
| 4 | F | 0.5 | 1183T>C | C395R | 0 | NA | expired |
| 5 | F | 3 | 90T>G | C31G | 2 | 48 | expired before receiving treatment |
| 6 | M | 120 | 1243G>A  386G>C | A415T  W129S | 0 | 22.5 | expired |
| 7* | M | 24 | 386G>C | W129S | 0 | 52 | expired |
| 8 | M | 14 | 1018G>A | D340N | 3 | 23 | expired |
| 9 | M | 240 | 1471G>A | D491N | 1 | 36 | expired |
| 10 | M | 3.5 | 658G>C | G220R | 0 | NA | expired |
| 11 | M | 3 | 528_529delinsAA | C176X | 1 | 16 | expired |
| 12 | M | 5 | 528_529delinsAA | C176X | 0 | 12 | expired |
| 13 | F | 0.75 | 1288insG | N457fsX28 | 0 | 22 | expired |
| 14 | F | 43.2 | 673C>T | R225W | 0 | NA | expired |
| 15* | F | 16 | 90T>G | C31G | 2 | NA | expired |
| 16* | F | 3 | 913G>A | G305S | 11 | NA | well post-BMT |
| 17 | M | 192 | 658G>A  1122G>A | G220S  W374X | 2 | NA | lost to follow up |
| 18 | M | 7 | 307_308insA | P336fsX121 | 4 | NA | expired |
| 19 | M | 384 | 694C>T | R232C | 7 | NA | expired |
| 20 | M | 108 | 148G>A | V50M | 2 | NA | expired |
| 21* | F | 3 | 1122G>A | W374X | 4 | 32 | lost to follow up |
| 22 | F | 18 | 148G>A | V50M | 2 | 36 | expired |
| 23 | M | 2 | 386G>C | W129S | 0 | NA | expired before receiving treatment |
| 24 | M | 8 | 82C>T | R28C | 6 | NA | lost to follow up |
| 25 | F | 4 | 1018G>G/A | D340N | 2 | NA | expired |
| 26 | M | 108 | 1349C>T | T450M | 2 | NA | expired |
| 27 | M | 0.75 | 916G>T | G306C | 2.5 | 8 | expired before receiving treatment |
| 28 | M | 10 | 510_510delC | S170Rfs*89 | 6 | NA | expired |
| 29 | F | 60 | 771C>A | C257X | 2 | NA | expired |
| 30 | M | 30 | 647T>C | I216T | 0 | 66 | expired before receiving treatment |
| 31 | M | 2 | 781A>G | E261k | 1 | 56 | expired before receiving treatment |
| 32* | M | 0.25 | 921delT | H308TfsX22 | ND | ND | lost to follow up |
| 33 | M | 2 | 1288G>T | D430Y | 0 | 62 | expired |
| 34 | F | 72 | 136G>A | E46K | 68 | 12 | expired |
| 35 | M | 180 | 1349C>T | T450M | 6 | 86 | on follow up, well |
| 36 | F | 48 | 386G>C  1349C>T | W129S  T450M | 5 | 5 | BMT planned |
| 37 | M | 2 | 528_529delinsAA | C176X | 0 | Normal | expired |
| 38 | F | 7 | 490C>T | C176X | 0 | ND | expired |
| 39* | M | 144 | 382G>G/A | D128N | 2 | abnormal | expired |
| 40* | M | 4 | 1574_1575del AA | E525GfsTer23 | <10 | NA | expired |
| 41 | M | 168 | 1519G>T,  1349C>T | E507ter,  T450M | 88.9 | NA | lost to follow up |
| 42 | F | 36 | 1349C>T,  658G>C | T450M,  G220R | NA | NA | lost to follow up |
| 43 | M | 72 | 673C>T | R225W | ND | ND | expired |
| 44 | M | 120 | 673C>T | R225W | ND | ND | well, No HLH Reactivation |
| 45 | M | 1 | NA | NA | NA | NA | expired post-BMT |
| 46 | F | 5 | NA | NA | NA | NA | expired post-BMT |
| 47 | F | 5 | NA | NA | NA | NA | expired post-BMT |
| 48 | M | 5 | NA | NA | NA | NA | well post-BMT |
| 49 | M | 2 | NA | NA | NA | NA | well post-BMT |
| 50 | M | 2 | NA | NA | NA | NA | well post-BMT |
| **FHL3** |  |  | ***UNC13D*** |  |  |  |  |
| 51 | M | 2 | 3031dupG | A1011GfsX15 | 83 | 2 | expired |
| 52* | F | 30 | 1072A>G | S58G | 92 | 2 | expired |
| 53 | M | 24 | 2722insACCT | S908Y fsX3 | 92 | NA | expired |
| 54 | M | 2 | 1772C>T | P591L | 85 | NA | expired |
| 55 | M | 3 | 2276dupG | V760RfsX29 | 73 | NA | expired post-BMT |
| 56 | F | 27 | 430insG | V144GfsX50 | NA | 22 | expired |
| 57 | M | 60 | 2599A>G  2448-8dupC | K867E  intronic | 94 | 10 | expired |
| 58 | M | 5 | 858+1G>A  2448-13G>A | splice site | 76 | 7 | well post-BMT |
| 59 | M | 1 | 2441delT | F814SfsX5 | 90 | 18 | expired |
| 60 | M | 1 | 1204C>T  1844A>G | L402V  D615G | 67 | 10 | asymptomatic and well |
| 61 | F | 2 | 1822del  640C>T | V608CfsX16  R214X | 96 | 17 | well till last follow up, now lost to follow up |
| 62 | F | 2.5 | 858+1G>A | splice site | 87 | 32 | expired |
| 63 | F | 4 | 2692delA | S898AfsX17 | 96 | 43 | lost to follow up |
| 64 | F | 72 | 2570T>C | F857S | 86 | 11 | expired |
| 65 | F | 24 | 762delC | C255AfsX73 | NA | NA | lost to follow up |
| 66 | M | 12 | 3031del | A1011PfsX18 | 95 | 3 | lost to follow up |
| 67 | F | 4 | 2625+1G>A | splice site | 88 | 5 | expired |
| 68 | M | 2 | 2819delT | L940RfsX13 | 97 | 3 | expired |
| 69 | F | 48 | 762del  1822del | C255AfsX73  V608CfsX16 | 96 | 54 | BMT planned |
| 70 | M | 12 | 3053C>A | A1018D | NA | NA | expired |
| 71 | M | 1 | 2569_2593delTTCCA CGCTGAGGGCTGTGG CCTGC | F857HfsTer50 | 87 | NA | expired |
| 72 | M | 396 | 1240C>T | R414C | NA | NA | on Phone Follow up |
| 73 | M | 4 | 858+1G>A  2448-13G>A | splice site | NA | 7 | well post-BMT |
| 74 | M | 11 | 381T>G  856C>T | D127E,  R286W | NA | NA | expired |
| 75 | M | 14 | 3031delG | A1011PfsTer18 | 95 | 2.80 | expired |
| 76* | M | 180 | 1204C>G | L402V | NA | NA | lost to follow up |
| 77* | M | 2.5 | 2482_2483insAG | V828EfsX20 | NA | NA | lost to follow up |
| 78* | M | 28 | 1204C>G | L402V | 84 | 5 | well |
| 79 | M | 12 | NA | NA | NA | NA | well post-BMT |
| 80 | F | 5 | NA | NA | NA | NA | well post-BMT |
| 81 | F | 6 | NA | NA | NA | NA | well post-BMT |
| 82 | M | 2 | NA | NA | NA | NA | well post-BMT |
| 83 | M | 6 | NA | NA | NA | NA | expired post-BMT |
| 84 | M | 9 | NA | NA | NA | NA | well post-BMT |
| 85 | F | 3 | NA | NA | NA | NA | well post-BMT |
| **FHL4** |  |  | ***STX11*** |  |  |  |  |
| 86 | F | 8 | 221C>T | T74M | NA | 7 | expired |
| 87 | M | 12 | 404T>C | L135P | 62 | 3 | expired |
| 88 | M | 96 | 772C>T | Q258X | 78 | 8 | lost to follow up |
| 89 | M | 3 | 418delC | Q140SfsX12 | 85 | 1.4 | expired |
| 90 | M | 18 | 173T>C | L58P | 92 | 12 | BMT planned |
| 91 | M | 60 | 173T>C | L58P | NA | NA | BMT planned |
| 92 | M | 144 | NA | NA | NA | NA | well post-BMT |
| **FHL5** |  |  | ***STXBP2*** |  |  |  |  |
| 93 | M | 132 | 1247-1G>C | splice site | NA | 11 | expired |
| 94 | M | 4.5 | 1697G>A | G566D | 92 | NA | expired before receiving protocol |
| 95 | M | 48 | 1247-1G>A | splice site | 92 | NA | well post-BMT |
| 96 | F | 4 | 194G>A  1730G>A | R65Q  G577D | 79 | 6 | expired |
| 97 | M | 96 | 1247-1G>C | splice site | 91 | 10 | expired |
| 98 | M | 2 | 902+2del | intronic | 99 | 1 | expired |
| 99 | M | 168 | 1247-1G>C | intronic | NA | NA | BMT planned |
| 100 | M | 6 | 610G>C | A204T | NA | NA | on cyclosporine |
| 101 | M | 60 | 1718C>T | P573L | NA | NA | lost to follow up |
|  |  |  |  |  |  |  |  |

NA: Details not available

ND: Not done

BMT: Bone marrow transplant

*: indicates monoallelic mutations
